# Case Report of Invasive Salmonella Superinfection of an Ovarian Endometrioma in a Patient With Systemic Lupus Erythematosus

**DOI:** 10.1177/11795476251382042

**Published:** 2025-10-31

**Authors:** Bright Etumuse, Nangah Tabukum, Nathan Buhler, Otto Pantoja, Yvette F. Gordon

**Affiliations:** 1 University of Texas Medical Branch, John Sealy School of Medicine, Galveston, USA; 2 Obstetrics and Gynecology Faculty, UTMB, Galveston, USA

**Keywords:** infectious diseases, systemic lupus erythematosus, gynecology, radiology, obstetrics/gynecology

## Abstract

**Objective:**

To present a case of invasive salmonella superinfection of an ovarian endometrioma in a patient with systemic lupus erythematosus, most likely due to exacerbation of a prior infection due to immunocompromised status, and review of literature on the typical and atypical manifestation of salmonella infection.

**Design:**

Case report and mini-review. We used the CARE checklist when writing our report.

**Materials and methods:**

For diagnosis of Salmonella bacteremia, cultures were used. Matrix-assisted laser desorption/ionization time-of-flight (MALDI-TOF) was used to further diagnose the patient with *Salmonella enterica ssp. diarizonae.*

**Setting:**

University-based OBGYN service.

**Patient:**

A 40-year-old G3P3003 female, initially presenting with nausea, non-bloody emesis, watery diarrhea, subjective fevers, and extreme fatigue, was found to have positive cultures for salmonella infection. Cefepime was administered with subsequent marked improvement in symptoms. Patient represented 2 months later with abdominal pain and fever and was confirmed to have a salmonella-infected endometrioma.

**Intervention:**

Explorative laparotomy, right salpingo-oophorectomy, and left adnexal cyst drainage.

**Main outcome:**

Outcome and complication of surgical management of salmonella superinfection of an ovarian endometrioma.

**Result:**

Post-op, the patient experienced post-op fevers until POD7, when her symptoms began to improve significantly. Persistent fevers were attributed to surgical complications involving a cyst rupture during removal. The patient was subsequently discharged with a 6-week course of Ciprofloxacin 500 mg BID and suppressive amoxicillin PO 500 mg BID after completing the Ciprofloxacin regimen due to immunocompromised status.

**Conclusion:**

Assessment for metastasis to other organs is crucial in evaluating immunocompromised patients diagnosed with salmonella infections. Surgical management should be highly considered.

## Introduction

Salmonella, named after veterinarian Daniel E. Salmon, is a gram-negative, rod-shaped bacterium belonging to the Enterobacteriaceae family. Salmonella is typically transmitted fecal-orally, often via contaminated water and improperly cooked foods.^1^ The prevalence of salmonella is higher in regions marked by overcrowding, social instability, and inadequate sanitation. Immunocompromised individuals, such as those with HIV, the elderly, infants, organ transplant recipients, or individuals with malignancies, face a dual risk - an increased vulnerability to infections and the experience of more severe or atypical disease manifestations. For instance, there have been documented cases of salmonella causing second-trimester pregnancy loss in pregnant women who typically undergo transient immunity changes.^2^ Previously published case reports have shown salmonella infection invading organs, including the spine, spleen, liver, psoas muscle, and brain, but very few cases of infected endometriomas have been reported.

Salmonella primarily infects humans and is not transmitted from animals. Common sources of salmonella infections include poultry and eggs. According to the Centers for Disease Control and Prevention (CDC), the United States alone reports approximately 1.35 million cases of salmonella infection annually, leading to 26 500 hospitalizations and, unfortunately, around 420 deaths. In the United States, Salmonella has been identified as 1 of the 3 pathogens responsible for the highest rates of hospitalization and fatalities attributed to food poisoning; however, it has been historically underreported.^1,3^ Presently, infections account for more than $4 billion annually in medical costs in the United States.^4^

Within the Salmonella genus, 2 major species prevail: *Salmonella enterica* and *Salmonella bongori*. These 2 variants encompass a staggering array of over 2500 serotypes.^5^ Additionally, Salmonella is renowned for its remarkable ability to endure extended periods under exceptionally unfavorable conditions.^6^

Although salmonella infections are primarily associated with gastrointestinal manifestations, they have also been linked to diverse pathologies in patients, including endovascular, orthopedic, obstetric, and gynecologic issues. For instance, in young patients with systemic lupus erythematosus (SLE), *Salmonella enteritidis B* commonly causes septic arthritis.^7^ It has also been reported as a rare cause of pelvic inflammatory disease^8^ and, as seen in this case, infected endometrioma. A case of recurrent bacteremia related to an infected endometrioma will now be discussed.

## Case Presentation

**Figure 1.**
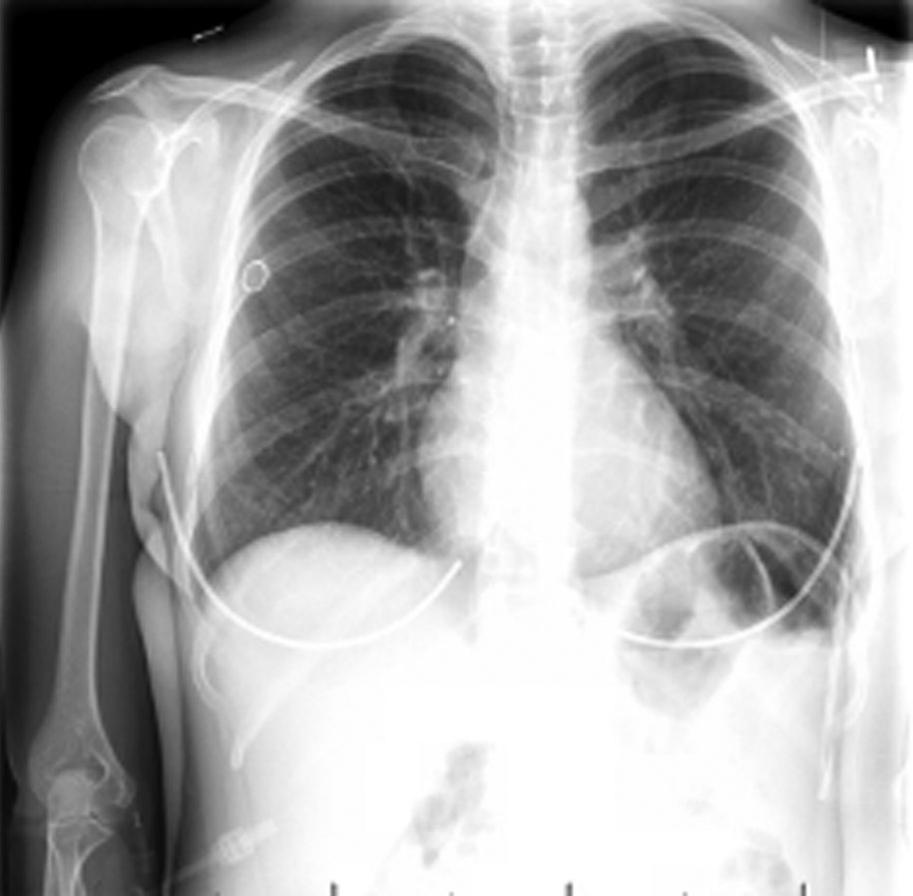
Initial presentation workup: XR chest showing punctate calcified granuloma at the left lower lung but no overall acute pulmonary process.

A Caucasian G3P3003 female in her early 40s with a past medical history of suspected but undiagnosed SLE presented to the ED with a 9-day history of nausea, non-bloody emesis, watery diarrhea, subjective fevers, and extreme fatigue. She has a long history of dysmenorrhea but no diagnosis of endometriosis. Family history is only positive for hypertension in her mother. She denied any sick contacts leading to her presentation. She tried over-the-counter cold medications and acetaminophen without improvement. Additionally, she noted bilateral finger discoloration indicative of Raynaud’s phenomenon, which she observed in the ED. She denied experiencing dysuria, headaches, syncope, chest pain, palpitations, shortness of breath, or lower extremity swelling. Her presentation raised suspicion for bacteremia given her high fevers. There was also suspicion of an autoimmune disorder such as SLE. Physical examination revealed alertness and orientation in 4 aspects—self, place, time, and current circumstances—yet she appeared lethargic and slow to respond to questions. Her vital signs were as follows: a maximum temperature of 103.4°F, blood pressure reading of 144/88 mmHg, a heart rate of 143 beats per minute (bpm), and oxygen saturation close to 100% on room air. Laboratory findings indicated leukocytosis, sodium level of 124 mmol/L, a D-dimer value of 1.97 µg/mL, pH of 7.47, and CO_2_ at 25 mmol/L, with negative results in the urinalysis and for COVID, Influenza, and RSV. Chest x-ray showed no signs of acute cardiopulmonary processes (Figure 1). Due to concerns about possible sepsis and suspected bacteremia due to the high fever and tachycardia, the patient received IV fluids and was admitted to the inpatient service.

**Figure 2.**
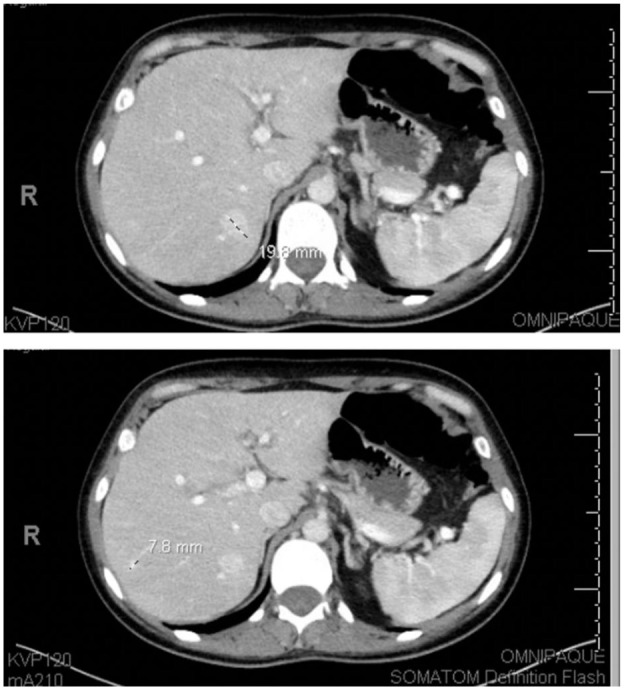
Initial presentation workup: CT abdomen pelvis showing two 19.8 and 7.8 mm right hepatic lobe enhancing lesions. Lesions were thought to represent a benign process, including hemangioma adenoma/FNH.

**Figure 3.**
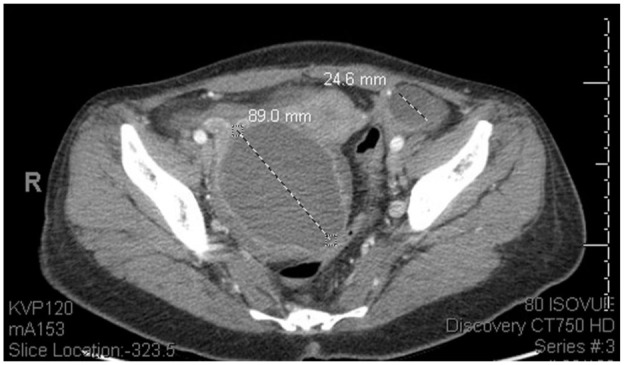
Initial presentation workup: CT abdomen pelvis showing an 8.9 cm complex cystic mass in the region of the right ovary and a 2.4 cm left ovarian cyst.

Once admitted, Cefepime was started and improvement in symptoms was noticed. Blood cultures were run, and about 2 days later, returned positive for Salmonella. The patient was then started on Ceftriaxone per susceptibilities. A computed tomography scan (CT) with contrast of the abdomen and pelvis was performed due to incomplete resolution of the patient’s symptoms and no known source of the infection. This CT showed 2 liver lesions, measuring 1.9 cm and 8 mm (Figure 2). An 8.9 cm complex cystic mass near the right ovary was also identified on the CT of the abdomen and pelvis (Figure 3). Rheumatology was consulted to evaluate bilateral finger discoloration. Autoimmune labs were anti-SM/SSA/SSB/dsDNA positive. Rheumatology recommended performing a TTE in order to evaluate for PAH in the setting of positive autoimmune labs. There was no evidence of pulmonary arterial hypertension on the transthoracic echocardiogram.

Ultrasound imaging showed an 8.9 cm structure in the right ovary with low-level internal echoes and a 3.3 cm structure in the left ovary suspected to be an endometrioma or a hemorrhagic cyst. These cysts were initially thought to be unrelated to her presentation, and a 6-week follow-up ultrasound with an outpatient OB-GYN was recommended to ensure resolution. She was diagnosed with salmonella bacteremia and SLE-related syndrome due to autoimmune labs. She was stabilized and discharged in fair condition with follow-up appointment instructions for outpatient OB-GYN and Infectious disease. Due to concerns of a liver abscess based on CT abdomen findings, she was placed on a 4-week course of Levaquin upon discharge. She was also discharged with Plaquenil 200 mg for SLE management.

Five weeks post-discharge, she reported to the infectious disease clinic for follow-up for salmonella bacteremia. In the clinic, she reported completion of her 4-week Levaquin prescription, stating her GI symptoms had entirely resolved. She denied any nausea, vomiting, diarrhea, abdominal pain, fever, chills, or any other associated symptoms. The patient’s salmonella bacteremia was proposed to result from ingesting contaminated food or water with an increased risk for invasive disease due to her underlying autoimmune condition. During a follow-up visit with rheumatology 1 week later, she was instructed to continue Plaquenil 200 mg daily, avoid cold exposure, and wear protective gloves.

Two months after discharge, the patient presented to the ED with complaints of abdominal pain and fever. She reported intermittent sharp, lower pelvic pain that began 5 days before her ED visit. She noted worsening and constant pain 3 days after onset. She stated the pain was almost debilitating and prevented her from getting out of bed. She also noted a subjective fever that began the day before the ED presentation. She used Tylenol for fever management and stated that her persistent symptoms, despite Tylenol 650 mg use, prompted her decision to report to the ED for evaluation. She also reported chills, 1 episode of watery diarrhea hours earlier, and decreased urine output with darker urine color. She denied any dysuria, hematuria, vomiting, or recent sick contacts.

Vital signs in the ED included blood pressure of 125/81 mmHg, pulse of 127 beats per minute, respirations of 18 per minute, temperature of 100.8°F, and SpO_2_ at 99% on room air. The patient was suspected to have pyelonephritis or ovarian torsion. Ultrasound (US) results showed low sonographic evidence for ovarian torsion, and urinalysis (UA) results negated any evidence of pyelonephritis. She received 1 g of IV Ceftriaxone, and a consult was requested for the OBGYN service.

**Figure 4.**
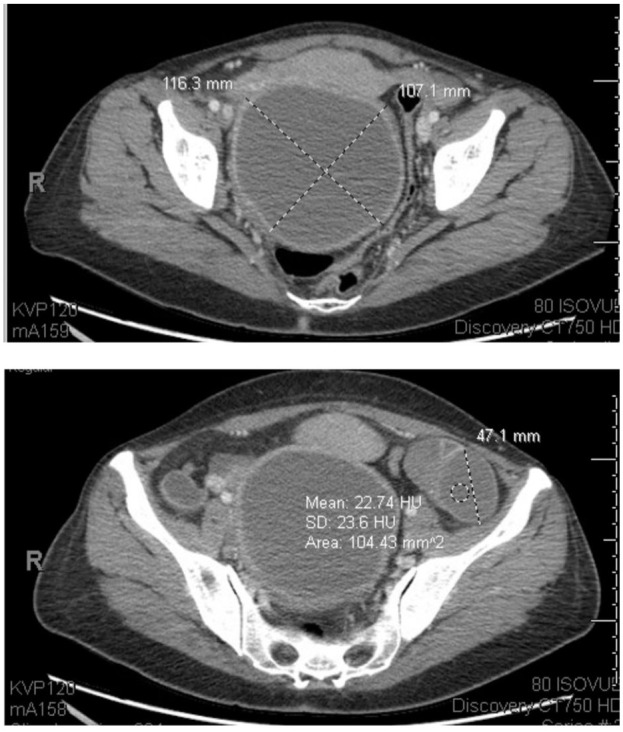
Second ED presentation workup: CT abdomen pelvis showing increased right adnexal cystic lesion with a thick enhancing wall measuring 11.6 cm × 10.7 cm × 10 cm compared to 10 cm × 8.7 cm × 8.6 cm during initial presentation. Multiple left ovarian hypoattenuating lesions, the largest measuring 4.7 cm, new compared to prior exam.

Upon OBGYN evaluation, she was found to have tenderness to light palpation in the bilateral lower quadrants, suprapubic tenderness, intermittent voluntary guarding, and a mass palpated in the right lower quadrant. On a bimanual exam, she was found to have a 10 cm firm palpable right adnexal mass with mass effect resulting in deviation of the cervix. No cervical motion or left adnexal tenderness was appreciated on exam. Rectovaginal exam confirmed right adnexal mass encompassing the posterior cul de sac; however, no nodularity was noted. Repeat imaging done in the ED showed an increase in the size of the right adnexal mass and new moderate right hydroureteronephrosis. The right adnexal mass measured 11.6 cm × 10.7 cm × 10 cm (Figure 4), increased from previous imaging (10 cm × 8 cm × 8 cm). Pelvic ultrasound also confirmed an increase in the right ovarian mass size consistent with an endometrioma, resulting in an anterior displacement of the uterus. She was subsequently admitted for overnight observation and pain control.

Labs ordered showed mildly elevated WBC at 12.09 × 10^3^/µL with left shift, lactic acid 1.14 mmol/L, negative urinalysis, and pending blood and urine cultures. Treatment with Ceftriaxone was continued, and complement levels, erythrocyte sedimentation rate, and C-reactive protein were ordered to rule out any autoimmune flare. Internal Medicine, Infectious Disease, and rheumatology were consulted. Lab results ordered include C-reactive protein 19 mg/dL, lactic acid 1.14 mmol/L, WBC 12 × 10^3^/µL, hemoglobin 11.3 g/dL, creatine kinase 50 U/L, C3 128 mg/dL, C4 12 mg/dL, and urinalysis with 30 mg/dL protein, no blood. With a C-reactive protein of 19 mg/dL, the Internal medicine team suspected mass effect versus infection and determined an SLE flare to be lower on the differential. Blood culture results returned positive for Salmonella species. Antibiotic treatment with Ceftriaxone was switched to piperacillin/tazobactam 3.375 g Q8H.

Due to the increased risk of salmonella-induced invasive disease into multiple organs, especially in immunocompromised hosts, the enlarged right adnexal cystic lesion was suspected to be the potential source of infection, and the infectious disease team recommended a surgical versus percutaneous sampling of the cystic lesions. The previously discovered liver lesions were also suspected to be a potential source of infection, and further CT abdomen imaging was recommended.

On hospital day 3, her fever and lower abdominal pain persisted despite continuous antibiotic treatment with IV piperacillin/tazobactam. The recurrent nature and persistence of the infection were suspected to be due to an endogenous source of infection. Since a transthoracic echo performed was unremarkable for vegetations and the liver lesions were smaller, hyperdense, and not increasing in size since the initial CT abdomen/pelvis, the enlarged ovarian mass was suspected as the infection source. IR culture of the adnexal mass ordered.

Due to unrelenting lower abdominal pain and non-resolving fevers despite IV antibiotic treatment, a decision to undergo an exploratory laparotomy, right salpingo-oophorectomy, and left adnexal cyst drainage for source control was made. A large 15 cm × 12 cm right adnexal mass was found and removed during the procedure. The mass ruptured during removal with 100 cc of purulent and chocolate cyst drainage. A 3 cm hemorrhagic cyst and a 4 cm endometrioma were punctured and drained from the left ovary. A normal appendix and normal liver surface were encountered during the surgery. Surgical samples were sent in for pathology evaluations and cultures.

**Figure 5.**
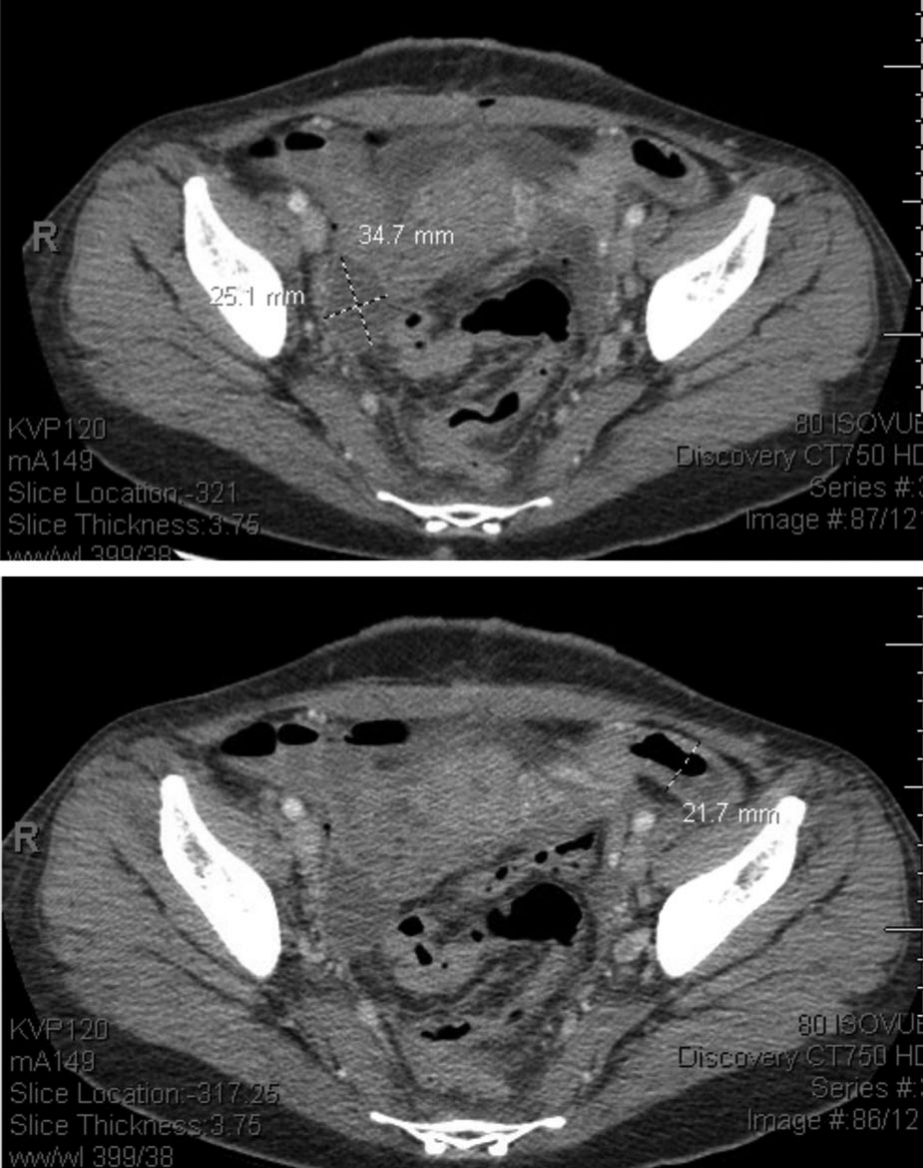
CT abdomen pelvis s/p exploratory laparotomy, right salpingo-oophorectomy with right adnexal mass removal, left adnexal mass drainage: right hemipelvic collection now measures 3.5 cm × 2.5 cm.

On post-op day (POD) 1, she reported some minor burning pain around her incision but otherwise good pain control with scheduled pain medications. On POD 2, she reported adequate pain control with PO meds and reported a new fever episode, which was attributed to a residual focus of infection or potentially new nidus of infection due to accidental cyst rupture in the OR. Nuclear Medicine imaging s/p surgery showed organized fluid collection in the right adnexa measuring 3.5 cm × 2.6 cm and was thought to represent early abscess formation or seroma (Figure 5). CT angiogram of the abdomen and pelvis showed a 3.3 cm × 3.9 cm ovoid area in the right adnexal region without a well-defined enhancing wall. Blood cultures were sent to the Texas Health Department in Austin, and the results returned positive for *Salmonella enterica* ssp. *diarizonae.*

On POD 4, she was found to have persistent fevers (*T*max 105.5°F) and was placed on IV Tylenol 500 mg in addition to PO Tylenol. IV piperacillin/tazobactam 3.375 Q6H was continued throughout the post-op period. NPO status was initiated at 0000 in the event of a possible drainage procedure. Repeat urine and blood cultures were ordered due to persistent signs of infection. On POD 5, piperacillin/tazobactam medication was switched to Ceftriaxone 2 g Q24H. On POD 6, she reported new onset brief episodes of tingling and numbness of the hand and legs, which she thought occurred when her antibiotic was changed to Ceftriaxone. On POD 7, she reported feeling significantly better. She reported adequate pain control with pain medications and denies any fevers, chest pain, shortness of breath, or any other associated symptoms. She was transitioned to Internal medicine as the primary service with continual OB/GYN follow-up.

**Figure 6.**
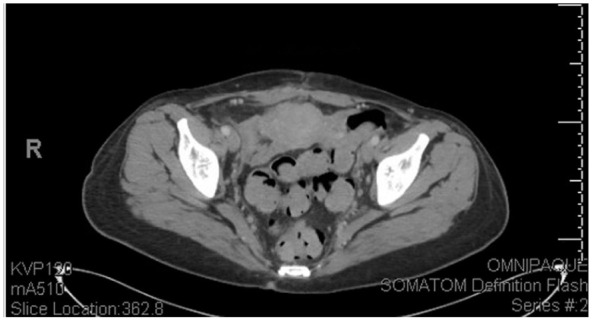
CT Abdomen Pelvis 26 days post-discharge: Interval resolution of right complex fluid collection now heterogenous granulation tissue and inflammation in the right adnexa but with no drainable fluid collection. New 3.3cm cm × 3.3 cm thin-walled cyst in the left adnexa associated with the left ovary. No pelvic free fluid. No inguinal or pelvic adenopathy.

On POD 8, she was found to be afebrile for the past 48 hours and had not received an antipyretic for the past 24 hours. On POD 9, she was finally discharged with a diagnosis of recurrent salmonella bacteremia secondary to an infected endometrioma with instructions to follow up with Infectious disease and OBGYN. She was discharged with a 6-week course Ciprofloxacin 500 mg BID therapy course and instructions to begin suppressive amoxicillin PO 500 mg BID after completing Ciprofloxacin therapy for another 3 months due to her immunocompromised status. A CT abdomen/pelvis was performed for follow up at 26 days discharge (Figure 6). A summary of the patient’s hospital stays can be seen in Table 1.

**Table 1.** A Timeline of Key Events During the Patient’s 2 Hospital Stays.

Date	Event
03/24/2022	Symptoms began (fever, fatigue, loss of appetite, sore throat, diarrhea, nausea, vomiting).
04/01/2022	Patient presented for the first time to the emergency department, cefepime and vancomycin started for high fever.
04/02/2022	CT abdomen/pelvis done with contrast. 8.9 cm pelvic mass discovered, believed to be an endometrioma. Plans for outpatient follow up with OB/GYN made.
04/04/2022	Blood cultures showing Salmonella. Susceptibility to ceftriaxone, cefepime/vancomycin changed to ceftriaxone. Symptoms improving.
04/08/2022	Patient discharged after first hospital stay. Symptoms resolved.
05/22/2022	Follow up CT abdomen/pelvis done, showing a cystic pelvic mass, still believed to be an endometrioma, now measuring 10 cm.
06/07/2022	Patient returns to the ED with fevers, abdominal pain, vomiting, diarrhea. CT of abdomen and pelvis done, right cystic pelvic mass now measuring 11.6 cm. Ceftriaxone initiated.
06/08/2022	Antibiotics changed to piperacillin-tazobactam due to infectious disease recommendation. Initial plan was to treat infection and then remove cystic pelvic mass outpatient at a later date after discharge.
06/14/2022	Plans made to remove the cystic pelvic mass due to lack of improvement with antibiotics and pain being localized to the mass.
06/15/2022	Exploratory laparotomy with right salpingo-oophorectomy, removal of 15 cm × 12 cm endometrioma.
06/24/2022	Symptoms resolved, patient discharged.

Source: Created by the authors.

### Timeline

## Discussion

In this case report, we describe the case of a metastatic spread of salmonella infection to an endometrioma in a 40-year-old female with no significant past medical history prior to presentation. This case report seeks to highlight important considerations in managing salmonella infections. Previously published case reports on salmonella infections may indicate an increased risk of invasive disease, especially in immunocompromised individuals. Other extra-intestinal complications are rare but well-described in adults involving the spine, sacroiliac region, spleen, liver, psoas muscle, brain, and, very rarely, the ovaries.^9^

One case report published in 2019 confirmed an ovarian abscess infected by *Salmonella typhi* in a 14-year-old girl who was not previously sexually active, raising suspicion for hematogenous spread from a primary infection site.^10^ Upon exploratory laparotomy, the right ovary was noted to be enlarged and pus-filled. The culture studies from the pus yielded *Salmonella typhi*. In this case, the cultured organism was found to be susceptible to all tested antibiotics, including ampicillin, chloramphenicol, trimethoprim-sulfamethoxazole, gentamicin, cephalothin, ceftriaxone, ciprofloxacin, and nalidixic acid. The patient was, however, treated with IV ceftriaxone 1 g BID, cloxacillin 1 g four times daily, and metronidazole 500 mg TID for 5 days.

In this case, the mass was drained, and the ovary preserved, contrary to our case in which the ovary was removed. Depending on the clinical presentation, the patient’s desire for future children, and the surgical feasibility of removing the mass without removing the ovary, this is a potential fertility sparing option.

Another case report published in 2017 describes a 28-year-old female with pre-existing endometriomas found to be infected with salmonella.^11^ Specifically, this case was the first reported case of a superinfection with *Salmonella enterica* serovar Schwarzengrund. This patient was initially started on empiric IV ciprofloxacin and metronidazole. Due to susceptibilities from stool cultures, ciprofloxacin was discontinued, and she was started on IV ceftriaxone. An infected large left ovarian abscess was removed surgically, and cultures were performed on the fluid associated with the mass, confirming a group B salmonella-infected endometrioma. The patient’s symptoms gradually resolved post-operatively, and she was discharged to complete 2 weeks of Ceftriaxone.

In our patient, her initial presenting symptoms completely resolved after empirical treatment with Cefepime, susceptibility-determined treatment with ceftriaxone, and outpatient treatment with a 4-week course of Levaquin. The clinical course following re-admission to the hospital was complicated by a ruptured ovarian cyst while being surgically removed. We propose that this complication led to her persistent signs of infection despite treatment with IV piperacillin/tazobactam 3.375 Q6H, which was later switched to Ceftriaxone 2g Q24H. To the best of our knowledge, this is the first reported case of *Salmonella enterica ssp. diarizonaei*-infected endometrioma in an immunocompromised individual with SLE. It is important to recognize that this is a single case of an infected endometrioma causing recurrent infection, and thus there are limitations in the conclusions that can be drawn from this research.

These occurrences highlight the unexpected but significant possible implications of salmonella species on reproductive health. The management of SLE utilizes immunosuppressive treatments and biologics like azathioprine, methotrexate, and mycophenolate to attain low disease activity or achieve remission.^8^ While crucial for effective disease management, this therapeutic approach induces immunosuppression, heightening susceptibility to infections. Notably, there was no ongoing immunosuppressive treatment at the time of the patient’s presentation. However, it is essential to recognize the increased prevalence of ovarian cysts in individuals with SLE compared to the general female population. This, combined with the likelihood of extraintestinal infections, emphasizes recognizing and managing these unforeseen disease variations, particularly within immunocompromised populations.

## Conclusion

It is crucial to assess patients diagnosed with salmonella infections for metastasis into other organs, including those with ovarian abscesses or masses such as endometrioma. In patients re-presenting for admission with similar symptoms after a successfully treated case of *Salmonella enteritidis* infection, assessment for metastatic sites of infection should be highly considered. Additionally, surgical management should be highly considered, especially in select disease manifestations such as an infected endometrioma. The heightened likelihood of infections beyond the intestines and the emergence of gynecologic complications due to salmonella accentuate the importance of recognizing and managing these unexpected manifestations, particularly within immunocompromised populations. Future research could focus on defining the incidence and risk factors for infection of endometriomas, comparing outcomes between fertility-sparing and definitive surgical management, and determining whether prophylactic removal of large endometriomas could reduce the risk of persistent or recurrent infection in high-risk patients.

## References

[1] Bhandari J Thada PK DeVos E . Typhoid fever. StatPearls [Internet]. StatPearls Publishing; 2023. Updated 2022. https://www.ncbi.nlm.nih.gov/books/NBK557513/32491445

[2] Pejcic-Karapetrovic B Gurnani K Russell MS Finlay BB Sad S Krishnan L . Pregnancy impairs the innate immune resistance to Salmonella typhimurium leading to rapid fatal infection. J Immunol. 2007;179(9):6088-6096. 17947683 10.4049/jimmunol.179.9.6088

[3] Mead PS Slutsker L Dietz V , et al. Food-related illness and death in the United States. Emerg Infect Dis. 1999;5(5):607-625. 10511517 10.3201/eid0505.990502 PMC2627714

[4] Economic Research Service. U.S. Department of Agriculture. Last Updated March 02, 2023). Cost Estimates of Foodborne Illnesses. https://www.ers.usda.gov/data-products/cost-estimates-of-foodborne-illnesses/. Accessed 2024.

[5] Waldner LL MacKenzie KD Köster W White AP . From exit to entry: Long-term survival and transmission of Salmonella. Pathogens. 2012;1(2):128-155. 25436767 10.3390/pathogens1020128 PMC4235688

[6] Finn S Condell O McClure P Amézquita A Fanning S . Mechanisms of survival, responses and sources of Salmonella in low-moisture environments. Front Microbiol. 2013;4:331. 24294212 10.3389/fmicb.2013.00331 PMC3827549

[7] Katarzyna PB Wiktor S Ewa D Piotr L . Current treatment of systemic lupus erythematosus: a clinician's perspective. Rheumatol Int. 2023;43(8):1395-1407. 37171669 10.1007/s00296-023-05306-5 PMC10261264

[8] Valayatham V . Salmonella: the pelvic masquerader. Int J Infect Dis. 2009;13(2):e53-e55. ISSN 1201-9712. https://www.sciencedirect.com/science/article/pii/S1201971208014173 10.1016/j.ijid.2008.06.015 18829361

[9] Gordon MA . Salmonella infections in immunocompromised adults. J Infect. 2008;56(6):413-422. 18474400 10.1016/j.jinf.2008.03.012

[10] Getahun S A Limaono J Ligaitukana R , et al. Ovarian abscess caused by Salmonella enterica serovar Typhi: a case report. J Med Case Rep. 2019;13(1):303. 31551082 10.1186/s13256-019-2229-y PMC6760102

[11] Adelman MW Johnson JH Hohmann EL Gandhi RT . Ovarian endometrioma superinfected with Salmonella: case report and review of the literature. Open Forum Infect Dis. 2017;4(2):ofx048. 10.1093/ofid/ofx048 PMC5407207 28470024

